# The Plant Alkaloid Harmaline Blocks the Voltage-Gated Sodium Channel Na_v_1.7: A Study Using an Automated Patch-Clamp

**DOI:** 10.3390/ijms26104636

**Published:** 2025-05-13

**Authors:** Jörg Eisfeld, Marina Schumacher, Mirjam Krautwald, Stephan Wierschke, Lu Qin, Taoufiq Fechtali, Heinrich Brinkmeier

**Affiliations:** 1 Institute of Pathophysiology, University Medicine Greifswald, Martin-Luther-Str. 6, D-17489 Greifswald, Germanylqin@ukaachen.de (L.Q.); 2 Cytocentrics Bioscience GmbH, 18059 Rostock, Germany; stephan.wierschke@mail.de; 3 Laboratoire de Neurosciences, Université Hassan II Casablanca, Mohammedia 28806, Morocco; taoufiq.fechtali@gmail.com; 4 Institute of Physiology, University Medicine Greifswald, Friedrich-Ludwig-Jahn Str. 15a, D-17475 Greifswald, Germany

**Keywords:** harmaline, ranolazine, pain, Na_v_1.7, sodium channel, late currents, automated patch-clamp

## Abstract

The voltage-gated sodium channel Na_v_1.7 is essential for pain perception and is an interesting target for the development of pain-relieving substances. Here, we investigated whether the Na_v_1.7 channel is sensitive to harmaline, an alkaloid produced by the North African plant *Peganum harmala*. To this end, we used Chinese hamster ovary (CHO) cells expressing the human Na_v_1.7 channel and studied Na^+^ channel pharmacology with an automated patch-clamp technique. Cells stimulated with depolarizing voltage pulses responded with typical transient inward currents. The Na^+^ channel blocker ranolazine inhibited whole-cell currents in a concentration-dependent manner (IC_50_: 12.1 µM). Harmaline inhibited both peak and late Na^+^ currents. A complete block was achieved at 300 µM of harmaline, with half maximum inhibition occurring at 35.5 µM. In contrast to ranolazine, the effect of harmaline was voltage independent. Neither the current/voltage curves nor the steady-state inactivation curves were shifted in response to drug application (30 µM). We conclude that the plant alkaloid harmaline, which is used in traditional medicine in North Africa, is an effective blocker of the voltage-gated Na^+^ channel Na_v_1.7. Our results offer a rationale for the use of harmaline against certain pain syndromes and rise hopes for the development of a new class of anti-nociceptive drugs targeting Na_v_1.7.

## 1. Introduction

Harmaline is one of several alkaloids produced by *Peganum harmala*, a plant growing in semiarid environments. Harmaline and related alkaloids belong to the class of β-carbolines and are probably produced as bioinsecticidal [1] and antimicrobial substances [2]. *P. harmala* (Syrian rue) is one of the oldest plants used in traditional medicine. Seed powder and seed extracts have been applied for the treatment of asthma, hypertension, diabetes, lumbago and other types of chronic pain [3,4]. At elevated dosages, harmaline and related compounds are cytotoxic [5] and can cause, amongst others, neurological symptoms in humans, such as visual hallucinations, agitation, tremors and ataxia [6].

Due to its multifaceted effects in humans and animals, it is not surprising that harmaline affects cells and organs via numerous biological target molecules. The vasorelaxant activity of harmaline seems to be mediated by blocking voltage-dependent Ca^2+^ channels of vascular smooth muscle and by stimulation of endothelial nitric oxide (NO) synthesis [7]. The spasmolytic effects of harmaline, as tested with isolated trachea preparations, are partly related to the block of Ca^2+^ channels. The synergistic relaxing effects seem to be caused by actions on several G-protein coupled receptors [8]. A prominent effect of harmaline on the central nervous system (CNS) is its capability to induce tremors in several mammalian species [9,10]. The mechanisms of harmaline-induced tremors are partially understood and seem to involve the inhibition of Ca^2+^ currents and, indirectly, the inhibition of Ca^2+^-dependent K^+^ currents in neurons of the inferior olive (IO) [9,11]. Additionally, a recent study described a reduction in Ca^2+^ levels in medium spiny neurons of the striatum with harmaline [12]. In any case, the overactivity of IO neurons in the medulla oblongata and feedback with motor circuits in the cerebellum appear to be at least partly responsible for this type of tremor [9,11].

Not only ion channels are targets of harmaline; it is also known as an inhibitor of monoamine oxidases (MAOs). The fact that MAOs catalyze the oxidation of monoamine neurotransmitters, such as dopamine and serotonin, explains some of the neuropsychological effects of harmaline [4,13].

One of the frequently mentioned applications of *P. harmala* extract is its usage against pain syndromes. Both positive experiences from traditional medicine [6] and observations from animal studies [3,14,15] argue for the analgesic effects of *P. harmala* alkaloids. The fractionation of *P. harmala* alkaloids revealed that harmaline is the most active analgesic compound of the alkaloids [14]. Recently, a placebo-controlled clinical trial showed a positive outcome associated with peganum oil medication in patients suffering from knee osteoarthritis [16]. Though there is much evidence that harmaline has beneficial effects in cases of certain types of pain, little is known about its mechanisms of action and its target molecules in the nociceptive system. Both the central and peripheral effects of harmaline seem to be important [15].

Pain perception is a complex process involving peripheral nociception, information processing in the CNS and descending pain-control pathways. The complexity of pain perception makes it difficult to discover specific targets of pain-relieving pharmacological substances in vivo. However, recent progress in the analysis of rare pain syndromes in humans underlined the importance of certain ion channels for peripheral pain perception. Mutations in the gene *SCN9A* encoding the voltage-gated sodium channel Na_v_1.7 have been related to two rare forms of increased pain perception: inherited erythromelalgia and paroxysmal extreme pain disorder [17]. The Na_v_1.7 mutations are gain of function mutations causing overactivity of the sodium channel [18] leading to hyperexcitability of Na_v_1.7-expressing neurons. On the other hand, a loss of function in Na_v_1.7 leads to a congenital indifference to pain [17]. Thus, the voltage-gated Na^+^ channel Na_v_1.7 is expressed in peripheral nociceptive neurons and its function is absolutely essential for pain perception. This feature makes the Na_v_1.7 channel an interesting target structure for pharmacological pain management, and at the same time, a candidate for being involved in the attenuation of pain in response to the administration of medicinal plant alkaloids, such as harmaline.

The present study was designed to clarify whether the medicinal plant alkaloid harmaline is a blocker of Na_v_1.7. To this end, we used a Chinese hamster ovary (CHO) cell line expressing the human Na_v_1.7 channel and studied Na^+^ channel pharmacology with an innovative automated patch-clamp system [19]. The concentration/response relationship of the action of harmaline on Na_v_1.7 revealed that the channel could be more sensitive to harmaline than other voltage-gated ion channels tested so far. Some of these results have been presented at the 96th Meeting of the German Physiological Society [20].

## 2. Results

### 2.1. Characterization of Na_v_1.7 Currents and Inhibition by Ranolazine

**Figure 1:**
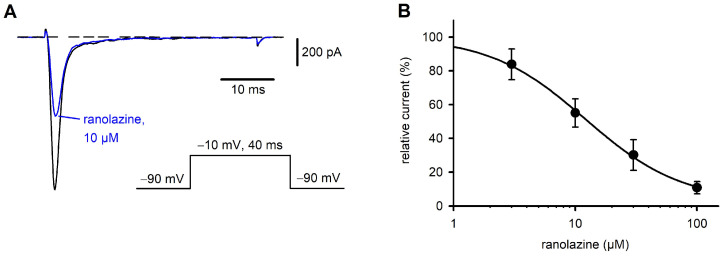
Cytopatch^TM^4 Mini as a reliable system for the study of drug effects. Whole-cell currents were recorded from CHO-K1 cells stably expressing the α-subunit of the human Na_v_1.7 channel. From a holding potential of −90 mV, the membrane was depolarized to −10 mV for 40 ms at a stimulation frequency of 1 Hz. (**A**) Representative current transients under control conditions (black) and after perfusion for 240 s with 10 µM of ranolazine (blue). (**B**) Concentration-dependent block of hNa_v_1.7. Between 4 (100 µM) and 9 cells were tested at each concentration. Data points were fitted by the equation f(x) = min + (max − min)/(1 + 10^(x-log IC^_50_^)h^) with x being the ranolazine concentration and h the Hill coefficient. An IC_50_ of 12.1 µM was calculated.

When CHO cells in the whole-cell configuration were stimulated with depolarizing voltage pulses to −10 mV, they responded with transient inward currents (Figure 1A, control trace). The kinetics of the currents were typical for voltage-gated Na^+^ channels and in agreement with the functional expression of the α-subunit of the Na_v_1.7 channel in the CHO cell line. During the first minutes of the recordings, the amplitudes of the Na_v_1.7 currents increased from values typically below 1 nA to 1–2 nA and then remained stable over time. Na^+^ inward currents could be completely blocked by the external application of 1 µM of tetrodotoxin (TTX, Figure S1A).

Ranolazine is an anti-anginal, local anesthetic-like drug that has been shown to block voltage-gated Na^+^ channels, including Na_v_1.7. To validate our cell system and the automated patch-clamp system, we studied the effect of ranolazine on whole-cell Na^+^ currents. Current transients were elicited by square voltage pulses going from −90 mV to −10 mV for 40 ms.

The application of 10 µM of ranolazine reduced the Na_v_1.7 current amplitudes by about 50% (Figure 1A). The effect occurred within several seconds and was reversible upon washout. The block of the Na_v_1.7 channel was dependent on the applied ranolazine concentration (Figure 1B). The calculated concentration required for half maximal inhibition (12.1 µM) agrees with previous results [21,22].

### 2.2. Effect of Harmaline on Na_v_1.7 Currents

**Figure 2:**
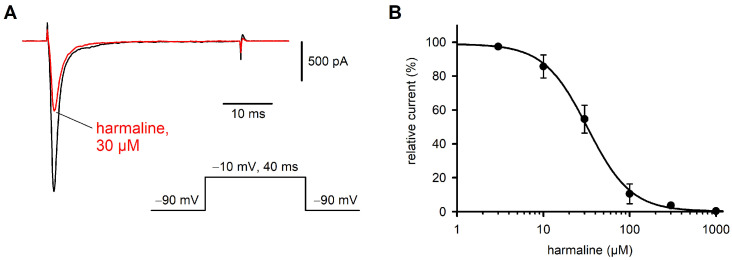
Concentration-dependent block of hNa_v_1.7 by harmaline. (**A**) Representative TTX-sensitive inward currents at −10 mV under control conditions (black) and after application of 30 µM of harmaline (red). Holding potential of −90 mV was applied throughout. (**B**). Harmaline at concentrations between 3 µM and 1 mM was continuously perfused until a steady state was achieved. Between 2 (1 mM of harmaline) and 11 cells were tested at each concentration. Average relative currents were plotted against harmaline concentration, a sigmoid curve was fitted to the data points (see legend to Figure 1) and an IC_50_ of 35.5 µM was calculated.

A similar series of experiments as shown with ranolazine was performed with harmaline. At a concentration of 30 µM, harmaline inhibited the Na_v_1.7 currents by about 50% (Figure 2). The effect of harmaline occurred within 1–3 min and was concentration dependent. A nearly complete block could be achieved at concentrations > 0.5 mM, while 5 µM was almost ineffective. From the concentration/response curve, a half maximal inhibitory concentration (IC_50_) of 35.5 µM was calculated for harmaline (Figure 2B). The channel kinetics seemed to be unaffected by harmaline, since normalized current transients recorded before and after drug application showed complete congruence in most cases (Figure S2).

**Figure 3:**
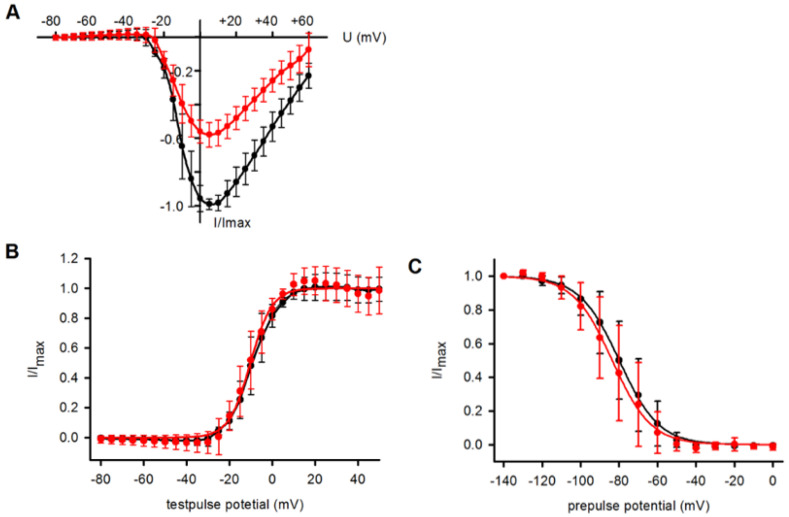
Voltage dependence of harmaline block. (**A**) Current/voltage (I/V) curves, generated from peak Na_v_1.7 currents, were first recorded in standard external solution and then in the presence of 30 µM of harmaline. Then, individual I/V curves obtained under control conditions were normalized between 0 and 1 and corresponding data in the presence of harmaline were calculated. Averaged normalized data points ± SD of n = 5 cells are shown for external solution (black) and 30 µM of harmaline (red). (**B**) Voltage dependence of activation of Na^+^ channels. Activation curves were calculated from I/V curves shown in (**A**). Boltzmann curves fitted to the data points resulted in half maximum activation of Na^+^ channels at −9.0 mV in external solution and −9.7 mV in the presence of harmaline. (**C**) Voltage dependence of inactivation of Na^+^ currents in the absence (black) and presence (red) of 30 µM of harmaline. Average normalized current maxima were plotted against prepulse potential. Data were obtained from n = 5 tested cells and Boltzmann curves fitted to the data points. Calculation of half maximal inactivation yielded on average −79.9 mV for the recordings in standard external solution and −83.5 mV in the presence of harmaline.

Many Na^+^ channel blockers, including ranolazine, induce a voltage-dependent block that can be shown by shifts in the steady state inactivation curve and/or the current/voltage curve of the Na^+^ currents. Harmaline blocked the Na^+^ currents of Na_v_1.7-expressing CHO cells without a significant influence on the position of the current/voltage curve (Figure 3A). Consequently, the normalized conductance/voltage curves were virtually congruent in the presence or absence of 30 µM of harmaline (Figure 3B). Boltzmann curves fitted to the data points revealed, on average, a half maximal channel activation at −9.0 mV in the standard external solution and −9.7 mV in the presence of 30 µM of harmaline. The slope factors for voltage dependence of activation were 6.1 in both conditions: standard external solution and harmaline-containing solution. The voltage dependence of the steady-state inactivation of the Na^+^ currents showed a tendency of a shift to more negative potentials by the drug (Figure 3C). Boltzmann curves fitted to the data points revealed a half maximal inactivation of Na_v_1.7 channels at −79.9 mV in the standard external solution (slope factor: 10.5) and −83.5 mV in the presence of 30 µM of harmaline (slope factor: 11.0).

To check for the specificity of the harmaline effect on Na_v_1.7 currents, we also tested the effect of the drug on the rat Na_v_1.2 channel and the human skeletal muscle Na^+^ channel (Na_v_1.4) expressed in HEK 293 cells. The latter experiments were performed with a manual path clamp [23]. The application of 30 µM of harmaline to the two latter cell lines inhibited voltage-gated Na^+^ currents by 18% (Na_v_1.4, n = 7 cells) and 11% (Na_v_1.2, n = 9 cells). The inhibition occurred in both cases within about 60 s and was widely reversible upon washout of the drug with the standard external solution (Figure S1).

### 2.3. Effect of Harmaline on Late Na^+^ Currents

**Figure 4:**
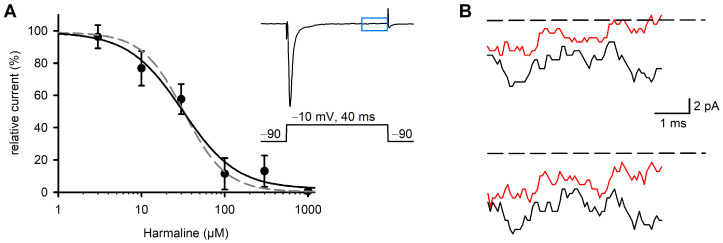
Effect of harmaline on Na_v_1.7 late currents recorded from CHO cells. From a holding potential of −90 mV, the membrane was depolarized to −10 mV for 40 ms at a stimulation frequency of 1 Hz. The last 5 ms of TTX-sensitive currents were analyzed for Na_late_; see inset in (**A**), blue box. (**A**) Harmaline, at concentrations ranging from 3 µM to 1 mM, was continuously perfused until a steady state was obtained. Then, 1 µM of TTX was applied and remaining nonspecific currents were subtracted offline. Relative currents were plotted against harmaline concentration, a sigmoid curve was fitted to the data points (black line) and an IC_50_ of 31.13 µM was calculated. Data points are given as means ± SD. For comparison, the concentration/response curve for inhibition of peak currents is superimposed (dashed grey line, see Figure 2B). (**B**) Representative TTX-sensitive inward currents (last five ms of original recordings, see inset in (**A**), blue box) at −10 mV under control conditions (black) and after perfusion with 30 µM of harmaline (red). Dashed line is the base line at 0 pA.

The Na^+^ inward currents recorded in response to 40 ms depolarizing voltage steps typically showed fast activating and inactivating phases, in addition to a late phase with the remaining constant channel activity. The amplitudes of these late currents were determined from the last 5 ms of the 40 ms traces. Their amplitudes reached about 0.1% of the peak currents of the same traces (Figure 4B). Late currents of the human Na_v_1.7 were inhibited by harmaline in a concentration-dependent manner. The concentration/response relationship yielded an IC_50_ of 31.1 µM (Figure 4A). In summary, the effects of harmaline on the late Na^+^ currents were almost identical to those on the peak currents. Shape and inflection points of the fitted concentration/response curves were identical (Figure 4A). The inhibition of Na_v_1.7 currents occurred within about 2–3 min and was widely reversible upon washout of the drug with the standard external solution (Figure S1).

## 3. Discussion

The plant alkaloid harmaline is considered the most valuable ingredient of *P. harmala* with respect to its pharmacological properties [4]. In the current study, we show that harmaline blocks the voltage-gated Na^+^ channel Na_v_1.7 in a concentration-dependent manner. Several important findings were derived. First, the harmaline concentration required to significantly inhibit whole-cell Na_v_1.7 currents was rather low compared to the effect of harmaline on other ion channels or electrophysiological and cellular functions. Second, the observed effect of harmaline on Na_v_1.7 channels is obviously not state-dependent, as is known about the effects of many other drugs and toxins on Na^+^ channels, as well as on voltage-gated Ca^2+^ channels. Third, we describe for the first time a peripheral molecular target of harmaline that could account for the pain-relieving properties of the drug in humans [3,4] and animal models [3] in vivo.

An early study related to this work has been presented by Splettstoesser and coworkers [24]. The authors used dorsal root ganglia (DRG) neurons from 3-week-old rats and investigated the effects of harmaline and harmane on voltage-gated channels. They found a half maximum inhibition of voltage-gated Ca^2+^ currents at 100 µM of harmaline, while the voltage-gated Na^+^ currents recorded from DRGs were reduced by less than 20% at that concentration. The application of 500 µM of harmaline reduced the Na^+^ currents by 80%, but preferentially in the voltage range > 40 mV. The authors concluded that voltage-gated Ca^2+^ channels of the L-/N-type were the most sensitive ones of the tested channels. They argued that the neuroprotective effects of harmaline could be related to its capability to block synaptic voltage-gated Ca^2+^ channels. A well-documented feature of harmaline is its ability to induce tremors by stimulating the neuronal activity of IO neurons in rats and mice [11,25]. In this context, ion channels seem to also be the molecular targets of harmaline. The harmaline concentrations required to achieve substantial effects on Ca^2+^ channels or cellular Ca^2+^ levels ranged between 125 µM and 250 µM [11] and between 62.5 µM and 125 µM [12], respectively. Only one study described substantial electrophysiological effects on Ca_v_1.3 channels at concentrations between 10 µM and 100 µM of harmaline [25].

Harmaline can also inhibit smooth muscle contraction, probably via its influence on Ca^2+^ channels [26]. The half maximum inhibition of induced contraction with noradrenaline in an isolated aorta model was achieved at 76 µM of harmaline. In the guinea pig taenia model, activated by carbachol, 70 µM (IC_50_) was required. In conclusion, the Na_v_1.7 channel seems to be a very sensitive molecular target of harmaline (IC_50_, 35.5 µM), at least among the cation channels tested so far. However, it should be considered that the voltage dependence of the channels and their pharmacological responsiveness may depend on the cell model, since the local membrane environment and intracellular factors are additional modulators of ion channels.

In contrast to the mechanism of block of Ca^2+^ channels by harmaline, we did not observe a state-dependent block of Na_v_1.7 channels. Neither the current/voltage curves of the Na^+^ currents nor the steady-state inactivation curves were shifted by harmaline. Half maximum inhibition of whole-cell Na_v_1.7 currents was achieved without significant shifts in the mentioned curves on the voltage axis (Figure 3). Many Na^+^ channel-blocking substances, such as drugs and toxins [27], exert their effects by shifting the inactivation curve to more negative potentials or shifting the activation to more positive potentials [28]. Both effects can reduce cellular excitability and preferentially reduce late currents. For harmaline, we observed a nearly identical inhibition of both peak and late currents (Figure 4B). Taken together, the mechanism of harmaline action on the Na_v_1.7 channel resembles that of substances occluding the channel pore [29] without noticeable voltage dependence of the block, at least as derived from this initial study.

The Na_v_1.7 is a peripheral target of harmaline and may explain the pain-relieving effects of the substance or the pain-relieving effects of ingredients of *P. harmala* and other sources of related β-carbolines. However, due to its numerous effects on the CNS, including neurotoxicity, the use of *P. harmala* ingredients for pain relief is not really recommendable. Furthermore, it is not known whether the harmaline concentration that is required for a significant influence on the action potentials of peripheral nociceptive neurons is achieved after the intake of harmaline in reasonable doses.

On the other hand, the Na_v_1.7 is sensitive to harmaline and peripheral nociceptive neurons are not shielded by the blood–brain barrier. Though harmaline is known to have significant effects on neuronal activity [9,25] and transmitter release in the CNS, its concentration in the rat brain after intravenous injection is much lower than that in the plasma samples of the same animals [30]. We suggest that harmaline concentrations in the µM range may inhibit the action potential generation of nociceptive neurons and thereby lead to pain relief in certain syndromes.

## 4. Materials and Methods

### 4.1. Cell Culture

CHO cells stably expressing the α-subunit of the human Na_v_1.7 (CHO-K1-Na_v_1.7) channel were obtained from Genomics AG (Zürich, Switzerland). The cells were grown in a medium composed of 90% DMEM/F12 (Gibco/Thermo Fisher Scientific, Darmstadt, Germany) and 10% fetal calf serum (Gibco/Thermo Fisher Scientific). To select for the expression of Na_v_1.7, the medium contained 500 µg/mL of hygromycin (InvivoGen, Toulouse, France). To prepare a cell suspension suitable for electrophysiological recordings from single cells, CHO cultures were washed with PBS and subsequently incubated with TrypLE^TM^ Express (Gibco/Thermo Fisher Scientific) for 5–10 min. Then, an excess of culture medium was added, and the cell suspension was gently mixed, centrifuged, washed twice and re-suspended in a solution composed of (in mM) 140 NaCl, 2.5 KCl, 2 CaCl_2_, 2 MgCl_2_, 10 HEPES and 19 Sucrose, at pH 7.4, adjusted to 320 mosm/L. The cell density was adjusted to 1–2 × 10^6^/mL. The obtained cell suspension could be stored in a cell reservoir for several hours. Some experiments were performed by manual patch-clamp [23]. For this, HEK 293 cells expressing the rat Na_v_1.2 or the human Na_v_1.4 channel were used. To select for Na^+^ channel-expressing cells, the medium contained 800 μg/mL of the antibiotic geneticin (G418, Gibco/Thermo Fisher Scientific). HEK 293 cells were grown in a medium composed of 90% MEM and 10% fetal calf serum (FCS; both from Gibco/Thermo Fisher Scientific).

### 4.2. Solutions and Drugs

The standard external solution for electrophysiological recordings contained the following (in mM): 140 NaCl, 2.5 KCl, 2 CaCl_2_, 1.2 MgCl_2_, 5 CsCl 10 HEPES and 5 D(+) glucose, at pH 7.4. The osmolality was adjusted to 320 mosmol/L with H_2_O or sucrose. The internal solution was composed of the following (in mM): 70 D-glucoronic acid, 70 CsOH, 60 CsCl, 10 NaCl, 1 CaCl_2_, 2 MgATP, 11 EGTA and 10 HEPES, at pH 7.2. The osmolality was adjusted to 290 mosmol/l. Harmaline and ranolazine dihydrochloride were obtained from Sigma-Aldrich (Steinheim, Germany) and tetrodotoxin (TTX) was obtained from Tocris Bioscience (Avonmouth, Bristol, UK).

### 4.3. Electrophysiological Recordings

Whole-cell Na^+^ currents were recorded at 22–23 °C using a Cytopatch^TM^4 Mini patch-clamp system (CytoBioscience, San Antonio, TX, USA), using standard dual-channel Cytocentrics chips with embedded quartz pipette tips of 2.5 µM diameter. The Cytopatch system is characterized by temperature stability, is equipped with a fast perfusion system and allows both current clamp and voltage-clamp configuration [19]. Pipette resistances yielded values of 3–4 MΩ when filled with standard internal solution. After the whole-cell configuration was established, currents were elicited by square voltage pulses going from a holding potential of −90 mV to −10 mV for 40 ms and a stimulation frequency of 1 Hz. During the first minutes of the recordings, peak inward currents often increased from values of about 0.5 to 1 nA to values between 1 and 2 nA and then remained stable. After a steady state was reached, pharmacological compounds were applied in a predefined sequence using the dispensing needle of automated equipment. All experiments were performed with continuous perfusion of the cell (10 µL/min). After 30 s of perfusion with 1 µM of Tetrodotoxin (TTX), the remaining nonspecific currents were subtracted offline.

To study the voltage dependence of activation of the Na^+^ channels, a cyclic pulse protocol was applied. Each cycle consisted of a 140 ms lasting prepulse to −120 mV to allow a substantial removal from inactivation of Na_v_1.7 channels. The prepulses were followed by 40 ms test pulses that were varied from −80 mV to +60 mV in 5 mV steps. The frequency of the depolarizing pulses was 1 Hz throughout the experiment. Peak inward currents and late Na^+^ currents were plotted and monitored over the entire duration of experiment. The amplitudes of the late currents were determined from the last 5 ms of the 40 ms traces. Late currents were calculated as the mean currents during the last 5 ms as the difference to the zero line.

To investigate the voltage dependence of inactivation of Na^+^ channels, another cyclic pulse protocol was applied consisting of a 140 ms conditioning pulse to −120 mV, followed by a 500 ms prepulse that was varied between −120 and +20 mV in 10 mV steps, and a 40 ms test pulse to −10 mV. Pulses were applied every 2.4 s. To obtain steady state inactivation curves, the peak currents recorded during the test pulses were plotted against the prepulse potential. Manual patch-clamp on HEK 293 cells was performed in the whole-cell configuration using an EPC 10 patch-clamp amplifier (HEKA Elektronik GmbH, Lambrecht, Germany), as recently described [23]. In the latter case, the preparation of cell suspensions (see above for Na_v_1.7-expressing cells) was not necessary. Suitable cells were manually picked from the bottom of the culture dishes using regular patch pipettes. All data are expressed as means ± standard deviation (SD).

## 5. Conclusions

We conclude that the voltage-gated Na^+^ channel Na_v_1.7 is a peripheral molecular target of the harmaline. A block of Na_v_1.7 channels in nociceptive neurons by harmaline may be related to the pain-relieving properties of the substance.

## Data Availability

All data are available from the corresponding author upon reasonable request. All data sets are presented as figures and are included within the manuscript.

## Supplementary Materials

The supporting information can be downloaded at https://www.mdpi.com/article/10.3390/ijms26104636/s1.

## Abbreviations

The following abbreviations are used in this manuscript:

CHO	Chinese hamster ovary cells
CNS	Central nervous system
DRG	Dorsal root ganglion
IO	Inferior olive
MAOs	Monoamine oxidases
Na_v_1.7	Voltage-gated sodium channel, α-subunit 9
NO	Nitric oxide
*SCN9A*	Gene, encoding the α-subunit of the voltage-gated sodium channel Na_v_1.7
TTX	Tetrodotoxin
