# Supplementary material for: The Plant Alkaloid Harmaline Blocks the Voltage-Gated Sodium Channel Nav1.7: A Study Using an Automated Patch-Clamp
Source: Int J Mol Sci. 2025 May 13;26(10):4636. doi: 10.3390/ijms26104636 (PMC12111501; doi:10.3390/ijms26104636)
Supplement: Supplementary file 1 [file ijms-26-04636-s001.zip › ijms-3604671-supplementary.pdf]

The plant alkaloid harmaline blocks the voltage-gated sodium channel $Na_v1.7$: a study using automated patch-clamp

Jörg Eisfeld, Marina Schumacher, Mirjam Krautwald, Stephan Wierschke, Lu Qin, Taoufiq Fechtali and Heinrich Brinkmeier

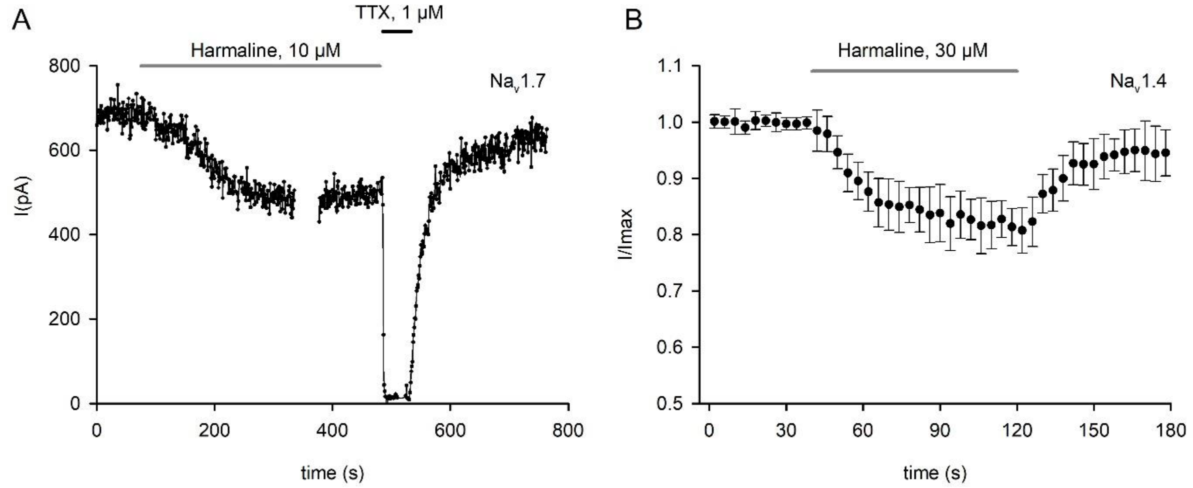

Figure S1. Time course of the harmaline action on sodium currents and reversibility of the effect. (**A**) Na^+ current transients were elicited in a $Na_v1.7$ expressing cell by depolarizing voltage pulses going from −90 mV to −10 mV for 40 ms. Peak Na^+ currents were plotted against the time (in s). At t = 70 s, harmaline was applied, causing an inhibition of peak Na^+ currents with a slow time course. In contrast, TTX caused a rapid and nearly complete bock of the channels. Washout of the drugs resulted in a slow revovery of the peak Na^+ currents. (**B**) Na^+ current transients were elicited in $Na_v1.4$ expressing cell by depolarizing voltage pulses going from −85 mV to −10 mV for 10 ms. Harmaline was applied between t = 40 s and t = 120 s. Peak current maxima were normalized and plotted against the time (in s). Means ± SD are given for all current maxima plotted (n = 6 cells tested).

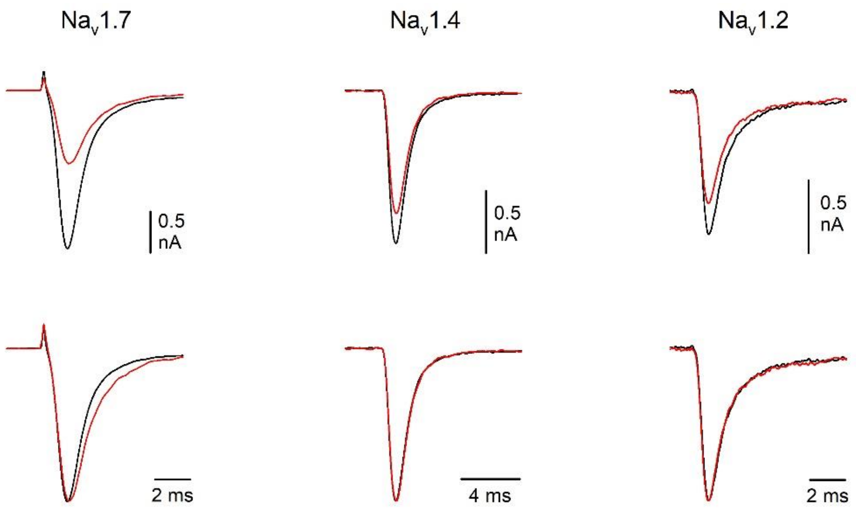

Figure S2. Sodium inward current transients in the presence (red lines) or absence (black lines) of harmaline. Concentrations of 10 μM (Nav1.7) and 30 μM (Na$_v$1.4 and Na$_v$1.2) were used, respectively. Inward current transients are only partially shown to illustrate fast activation and inactivation phases. Upper row: Current transients show in nA; lower row: corresponding normalized current transients.
